# A Prospective Case Series on the Effect of Cross‐Linked Hyaluronic Acid Combined With a Collagen Sponge in the Treatment of Alveolar Osteitis

**DOI:** 10.1002/cre2.70388

**Published:** 2026-08-10

**Authors:** Danijel Domic, Peter Bauer, Nicola Ottenbacher, Kristina Bertl, Christian Ulm, Andreas Stavropoulos

**Affiliations:** ^1^ Division of Oral Surgery University Clinic of Dentistry, Medical University Vienna Vienna Austria; ^2^ Department of Periodontology, Dental Clinic, Faculty of Medicine Sigmund Freud University Vienna Austria; ^3^ Department of Periodontology Blekinge Hospital Karlskrona Sweden; ^4^ Periodontology, Faculty of Odontology University of Malmö Malmö Sweden; ^5^ Division of Conservative Dentistry and Periodontology University Clinic of Dentistry, Medical University of Vienna Vienna Austria; ^6^ Department of Periodontology, School of Dental Medicine University of Bern Bern Switzerland

**Keywords:** alveolar osteitis, dry socket, hyaluronic acid, tooth extraction, wound healing

## Abstract

**Objectives:**

This case series reports on alveolar osteitis (AO) treatment outcomes after standard AO therapy combined with cross‐linked hyaluronic acid (xHyA) and a collagen sponge.

**Material and Methods:**

Nine patients fulfilling the AO criteria after permanent tooth extraction were included. After standard AO treatment, that is, irrigation and curettage, a xHyA gel combined with an absorbable collagen sponge was applied into the socket and stabilized with a suture. Pain perception (visual analogue scale [VAS], pain killer consumption), soft tissue healing index (STHI), alveolar bone exposure, necessity of re‐treatment, occurrence of complications, and patients’ opinion were assessed up to 14 days post‐treatment.

**Results:**

Eight patients completed the 14‐day follow‐up, while one patient dropped out after 1 day due to persisting pain. Initial mean pain perception (VAS) was 7.5 (1.4), which decreased after a single treatment to 4.4 (2.2) within 6 h and by 50% (3.8 [2.4]) within 24 h. In 5 out of 8 patients, complete pain resolution was achieved after 14 days, while 3 patients still experienced moderate pain. Pain reduction was accompanied by an improvement of STHI, reaching 4.0 (0.8) within 14 days; complete coverage of exposed alveolar bone walls was already observed within 3 days in all 8 patients. Only one patient required re‐treatment; 5 out of 8 patients expressed willingness to pay on top between 50 and 300€ for the material costs (xHyA+collagen sponge).

**Conclusions:**

A single application of xHyA combined with a collagen sponge offered effective and fast pain relief combined with a favorable healing process in patients suffering from AO. Furthermore, the need for re‐treatment was infrequent, and favorable patient feedback was observed. However, randomized controlled trials with a sufficient sample size and a control group are essential to validate these findings.

## 1 Introduction

Alveolar osteitis (AO) is a painful complication occurring in about 5% of the cases after standard tooth extraction (Daly et al. 2012). The term “AO” was introduced by Crawford in 1896, and numerous synonyms have been proposed, such as dry/septic/necrotic socket, localized osteitis, postoperative/fibrinolytic alveolitis, localized osteomyelitis, etc. (Chow et al. 2020). In 2002, Blum suggested the following definition for AO: postoperative pain in and around the extraction site that intensifies within 1 to 3 days after extraction and often extends to the temporal and/or auricular region. This pain is accompanied by a partially or completely disintegrated blood clot in the socket, with or without *fetor ex ore*, and cannot be attributed to other causes (Blum 2002). Clinically, exposed alveolar bone, sometimes covered by a yellow–gray necrotic tissue layer, and/or inflamed surrounding soft tissue is often observed. While the regular healing process after tooth extraction relies on the rapid formation and stability of a fibrin network, that, a blood clot, AO occurs due to premature loss of this blood clot. This can happen either due to excessive fibrinolysis (Nitzan et al. 1978), which is linked to poor oral hygiene, periodontitis, or pericoronitis (Rud 1970), or mechanical means (e.g., excessive use of a mouth rinsing solution). Several risk factors have been identified, such as mandibular extraction site, female gender, use of oral contraceptives, poor oral hygiene, smoking, and surgical tooth removal including raising a mucoperiosteal flap, tooth sectioning, and/or osteotomy (Eshghpour et al. 2015; Kolokythas et al. 2010; Veale 2015); for example, AO after surgical removal of lower third molars has an incidence beyond 30% (Daly et al. 2012; Fridrich and Olson 1990).

The standard treatment of AO includes removal of the debris, for example, by irrigation with sterile physiological saline solution, and debridement of the socket to induce bleeding and allow the formation of a new blood clot. In addition to standard AO therapy, several adjunctive methods have been assessed, ranging from direct application of dressings into the extraction socket (e.g., iodoform, blood‐derived concentrates, zinc oxide eugenol, or plant‐based alternatives, etc.) to the use of laser (e.g., low‐level laser) (Kamal, Salman, Abdul Razak, Qabbani et al. 2020; Kaya et al. 2011; Chaurasia et al. 2017). However, any evidence on the superiority of one of these adjuncts in comparison with standard AO treatment remains inconclusive (Kamal, Salman, Abdul Razak, Qabbani et al. 2020; Kamal, Salman, Abdul Razak, Samsudin et al. 2020; Ghosh et al. 2021).

In this context hyaluronic acid (HyA), which is a hydrophilic glycosaminoglycan naturally present in the human body, including in various oral fluids and tissues, such as saliva, gingival crevicular fluid, periodontium, alveolar bone, etc. (Bartold et al. 1988; Embery et al. 1982; Waddington et al. 1989), has been tested to prevent AO after non‐surgical (Bayoumi et al. 2015; Almushalbn et al. 2022; Domic et al. 2023) and surgical tooth removal (Munoz‐Camara et al. 2020; Afat et al. 2019; Guazzo et al. 2018; Domic et al. 2023) as well as an adjunct measure in the treatment of AO (Dubovina et al. 2016; Suchánek et al. 2019; Hakobyan et al. 2020; Khachatryan et al. 2025). For example, in one of the latter four studies, a self‐prepared, sponge‐like medical device containing octenidine dihydrochloride (OCDA) and HyA was used, which was re‐applied every 2 days until pain resolution; however, this study was lacking a control group (Suchánek et al. 2019). The other three studies reported either no additional effect of HyA on the pain level compared to the corresponding control group (Dubovina et al. 2016) or a significantly reduced pain when HyA was combined with magnetic laser therapy and compared either to magnetic laser therapy only (Hakobyan et al. 2020) or to standard treatment in patients with compromised healing (i.e., diabetes mellitus type 2) (Khachatryan et al. 2025).

However, HyA may exhibit different results depending on its molecular weight and cross‐linking status. For example, high molecular weight (hMW) HyA has been shown to improve wound healing compared to low and medium molecular weight HyA (Fouda et al. 2016; Knudson and Knudson 1993; David‐Raoudi et al. 2008; Stern et al. 2006; Pilloni et al. 2025). In addition, a cross‐linking process can improve the stability and reduce the resorption rate of HyA (Asparuhova et al. 2019; Burdick and Prestwich 2011; Pilloni et al. 2025). Nevertheless, up to now, the effect of cross‐linked HyA (xHyA) has not been assessed. Therefore, the aim of the present case series was to report on the AO treatment outcomes (i.e., patients’ pain perception, soft tissue healing, formation of granulation tissue) after standard AO therapy combined with xHyA and an absorbable collagen sponge.

## 2 Patients and Methods

### 2.1 Study Protocol and Registration

This prospective case series is reported in accordance with the PROCESS guidelines for reporting clinical case series (Appendix 1) (Agha et al. 2020), and the treatment protocol was approved by the Ethics Committee of the Medical University of Vienna (Austria; EC number: 1049/2021). All patients who presented at the University Clinic of Dentistry (Medical University of Vienna, Austria) and were diagnosed with AO were informed about the study and enrolled in this trial, after written informed consent was obtained.

### 2.2 Definition of AO and Eligibility Criteria

AO diagnosis was based on the definition of Blum (2002), and the following eligibility criteria were applied in the recruitment process: (1) Age ≥ 18 years; (2) AO developing after extraction of a permanent tooth (excluding wisdom teeth); (3) absence of systemic diseases (e.g., osteoporosis) and no intake of medications known to affect hard and/or soft tissue healing (e.g., bisphosphonates); (4) no untreated periodontitis; (5) no known hypersensitivity or allergy to the products used herein; (6) no pregnancy; and (7) willingness to attend the scheduled follow‐up appointments.

### 2.3 Treatment of AO

**Figure 1:**
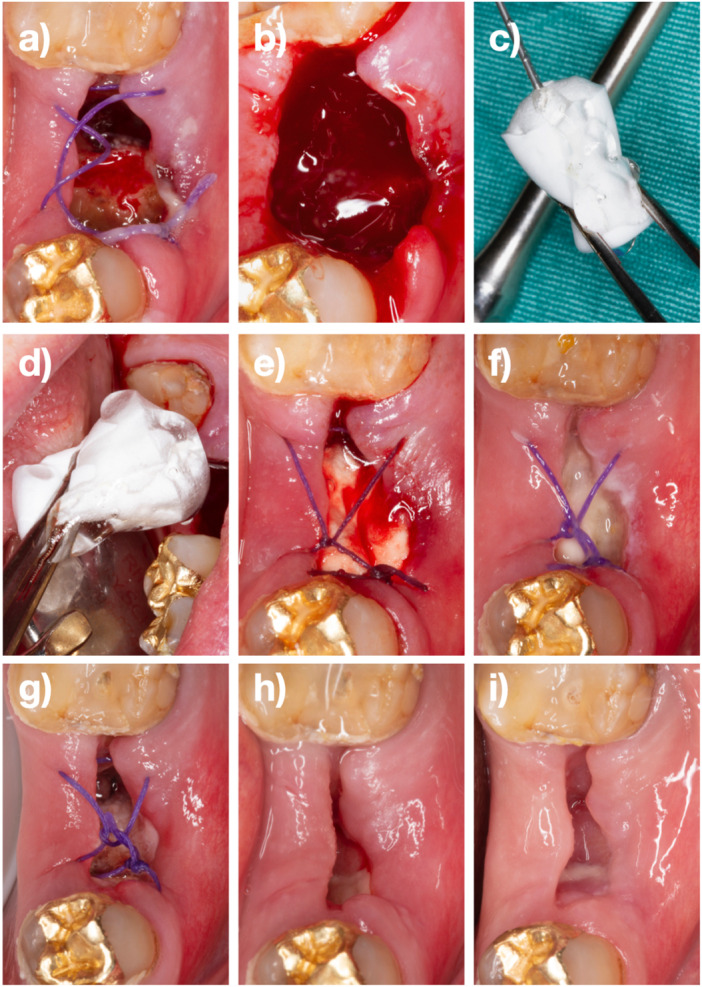
Patient #7 developed AO 3 days after extraction of a lower first molar (tooth #36); (a) AO diagnosis, (b) standard AO therapy including induction of bleeding and direct application of the xHyA gel into the extraction socket, (c) xHyA gel was additionally injected into a collagen sponge, which was (d) applied into the extraction socket, (e) application of a simple cross suture, and follow‐up appointments after (f) 1, (g) 3, (h) 7, and (i) 14 days.

Management of AO followed a standard clinical protocol. Local anesthesia was administered (inferior alveolar nerve block and/or buccal and lingual terminal infiltration) using local anesthesia without adrenaline (Ultracain Dental, Sanofi‐Aventis GmbH, Vienna, Austria). Subsequently, the extraction socket was thoroughly irrigated with sterile saline solution, and all debris was carefully removed. With a surgical curette, bleeding was induced by scratching the bony walls of the socket. In addition to this standard procedure, the extraction sockets were filled with a hMW HyA gel containing 1.6% xHyA and 0.2% non‐xHyA (HyaDENT BG, Regedent AG, Zürich, Switzerland) and an absorbable collagen sponge (PARASORB Cone, RESORBA Medical GmbH, Nürnberg, Germany). More specifically, the xHyA gel was injected outside of the mouth into and around the collagen sponge and left for a few minutes to allow saturation of the sponge with the xHyA gel. The xHyA gel was additionally applied directly into the extraction socket prior to application of the xHyA‐collagen sponge. Finally, a modified horizontal mattress suture was placed (Vicryl 4‐0, Ethicon, Somerville, USA), and patients were instructed to bite on a gauze for at least 30 min to optimize stabilization of the blood clot (Figure 1a–e). All procedures were performed by one of 2 experienced oral surgeons (D.D. and P.B.). The following postoperative instructions were given to the patient: (1) physical protection and refraining from sports for 7 days; (2) cooling of the treated site for 2 to 3 days; (3) refraining from any mouth rinsing solution; and (4) prescribed medication intake (ibuprofen 400 mg or paracetamol 500 mg, 3 times per day for 2 days and thereafter as required). Suture removal was scheduled 7 days post‐intervention.

### 2.4 Clinical Parameters and Follow‐Up Scheme

The following patient characteristics were collected: (1) gender; (2) age; (3) smoking status, including number of cigarettes; (4) presence of systemic diseases; and (5) medication intake. Regarding smoking, pack‐years were calculated as the number of cigarettes smoked per day divided by 20 and multiplied by the total number of years smoking.

The following site/treatment‐specific data were recorded prior to, during, and after AO treatment:

1. reason for tooth extraction and position of the extracted tooth;
2. patients’ pain sensation was measured with (a) a 10 cm visual analog scale (VAS) and categorized as following: “no pain” (VAS 0 < 0.2), “mild pain” (VAS ≥ 0.2 < 1.7), “moderate pain” (VAS ≥ 1.7 < 4.7), “severe pain” (VAS ≥ 4.7 < 7.7), “very severe pain” (VAS ≥ 7.7 < 9.6), and “most severe pain imaginable” (VAS ≥ 9.6–10) (Aicher et al. 2012); pain assessment was performed prior to, 6 and 12 h as well as 1, 2, 3, 4, 5, 6, 7, and 14 days after AO treatment; and (b) by recording pain killer consumption (i.e., number of tablets and days after AO treatment);
3. soft tissue healing index (STHI) was assessed prior to AO treatment and 1, 3, 7, and 14 days after AO treatment (Turnbull et al. 1986); STHI was graded as following: “1” very poor, with ≥ 50% of the gingiva being red, positive bleeding on palpation and suppuration; “2” poor, with ≥ 50% of the gingiva being red, positive bleeding on palpation, but without suppuration; “3” good, with ≥ 25% but < 50% of the gingiva being red and negative bleeding on palpation; “4” very good, with < 25% of the gingiva being red and negative bleeding on palpation, and “5” excellent, when all tissues are pink and negative bleeding on palpation. The STHI was slightly adapted for the purpose of the present study, as wound palpation was performed using dental tweezers, and any evaluation of the “incision margin” was excluded due to secondary intention healing of the extraction sockets;
4. integrity of the extraction socket recorded as the number of missing bone walls from 1 to 4 at the time of AO treatment;
5. alveolar bone exposure evaluated prior to AO treatment and 1, 3, 7, and 14 days after AO treatment which was graded as following: “0” no exposed bone wall, “1” one exposed bone wall, “2” 2 exposed bone walls, “3” 3 exposed bone walls, and “4” all 4 bone walls exposed (Sharma et al. 2017);
6. necessity of re‐treatment, which was performed at the earliest 3 days after initial AO treatment;
7. presence of any other complication, such as appearance of a bone sequestrum; and
8. willingness to pay for the treatment assessed with the following 2 questions (Q) at the last follow‐up after 14 days: Q1: “If the treatment with HyA would indeed reduce your pain faster than the standard treatment, would you be willing to pay for it?” and Q2: “If yes, how much would you be willing to pay?”

### 2.5 Statistical Analysis

Clinical outcomes were summarized descriptively using means and standard deviations (SD) as well as medians and interquartile range. Due to a small sample size and lack of a control group, no inferential statistical analysis was performed. Individual case details were presented to illustrate variability and clinical observations across patients.

## 3 Results

### 3.1 Patient Demographics and Baseline Characteristics

Between July 2021 and July 2024, 9 patients (6 female, 3 male) with a mean age of 46.8 (15.2) years were enrolled; 3 patients were smokers, and 5 patients presented with ≥ 1 systemic condition. A total of 8 patients were diagnosed with AO in the mandible, and 1 patient had received standard AO treatment (i.e., surgical curettage of the extraction socket) 2 days prior to study inclusion, but did not show any improvement; 7 out of 9 patients presented with a fully intact extraction socket, while in 2 patients 1 bone wall was missing. For details, see Table 1.

**Table 1:** Characteristics and treatment outcomes of the included patients. The coloring of the pain levels (VAS) corresponds to the legend presented in Figure 2.

		Patient 1	Patient 2	Patient 3	Patient 4	Patient 5	Patient 6	Patient 7	Patient 8	Patient 9	Summary
Gender		Female	Male	Female	Female	Female	Male	Female	Male	Female	6 Female/3 Male
Age		61	31	41	65	36	40	73	38	36	46.8 (15.2)^a^
Smoking	Cig/d	0	2	20	0	0	0	0	1	0	7.7 (10.7)^a^
	Pack‐years	0	1.9	20	0	0	0	0	0.7	0	7.5 (10.8)^a^
Systemic disease		Osteoarthritis, hip prosthesis	None	Crohn's disease	Hypercholesterolemia, hypertension, chronic bronchitis, asthma	None	None	Hypertension, hypothyroidism, lung transplant	None	Hypothyroidism	5/9^b^
Extraction site		16	36	44	36	37	35	36	46	36	2 Premolars/7 Molars
Previous AO therapy		No	No	No	No	No	No	No	No	Yes	1/9^b^
Integrity of the extraction socket		4	4	4	4	3	4	3	4	4	3.8 (0.4)^a^
**Pain (VAS, 0–10)**											
Preoperative		8.5	8.8	8.0	7.6	7.5	7.3	8.7	7.0	4.3	7.5 (1.4)^a^
6 h		7.9	1.6	5.0	3.3	6.2	NR	NR	4.1	2.5	4.4 (2.2)^a^
12 h		7.0	6.0	5.4	0.6	6.3	NR	NR	2.7	2.0	4.3 (2.5)^a^
Day 1		6.2	5.0	2.0	1.5	7.5	6.3	1.7	1.9	2.0	3.8 (2.4)^a^
Day 2		Drop‐out	5.9	3.8	3.2	6.0	NR	NR	3.0	3.5	4.2 (1.4)^a^
Day 3			5.0	0.5	1.5	6.0	1.7	0	6.0	1.6	2.8 (2.5)^a^
Day 4			2.3	2.4	2.0	5.7	NR	NR	7.1	1.2	3.5 (2.4)^a^
Day 5			5.2	2.8	1.7	5.1	NR	NR	1.9	0.5	2.9 (1.9)^a^
Day 6			2.0	1.0	0.5	4.8	NR	NR	0.6	0.4	1.6 (1.7)^a^
Day 7			1.6	0.5	0	4.0	0.2	0	0.7	0	0.9 (1.4)^a^
Day 14			3.5	0	0	3.0	0	0	0	4.1	1.3 (1.9)^a^
**Painkillers (*n***)^c^											
Day 1		4	3	3	1	3	3	3	3	0	2.6 (1.2)^a^
Day 2		Drop‐out	3	3	1	3	3	3	3	0	2.4 (1.2)^a^
Day 3			1	0	1	1	2	2	1	0	1.0 (0.7)^a^
Day 4			2	1	0	3	2	0	2	0	1.3 (1.1)^a^
Day 5			1	2	0	2	1	0	3	0	1.1 (1.1)^a^
Day 6			0	1	0	2	0	0	2	0	0.6 (0.9)^a^
Day 7			0	0	0	0	0	0	0	0	0^a^
Day 14			0	0	0	0	0	0	0	0	0^a^
Total		4	10	10	3	14	11	8	14	0	8.2 (4.9)^a^
**STHI (1–5)**											
Preoperative		3	3	1	1	2	2	1	2	3	2.0 (0.9)^a^
Day 1		2	2	2	3	2	4	3	3	4	2.8 (0.8)^a^
Day 3		Drop‐out	3	4	3	2	4	4	NR	4	3.4 (0.8)^a^
Day 7			3	3	4	3	4	5	3	4	3.6 (0.7)^a^
Day 14			3	4	4	3	NR	5	5	4	4.0 (0.8)^a^
**Bone exposure (0–4)**											
Preoperative		1	0	4	2	0	4	4	3	2	2.2 (1.6)^a^
Day 1		0	0	4	0	3	0	0	0	0	0.8 (1.6)^a^
Day 3		Drop‐out	0	0	0	0	0	0	NR	0	0^a^
Day 7			0	0	0	0	0	0	0	0	0^a^
Day 14			1	0	0	0	0	0	0	0	0.1 (0.4)^a^
Necessity of re‐treatment		No	No	No	No	No	No	No	Yes (after 3 days)	No	1/9^b^
Complications		No	No	No	No	No	No	No	No	Yes (bone sequestrum)	1/9^b^
Willingness to pay (yes/no, €)		Drop‐out	No	50–100€	300€	No	100€	> 150€	80€	No	5/8^b^

Abbreviations: AO, alveolar osteitis; Cig/d, cigarettes per day; h, hour; n, number; NR, not reported; STHI, soft tissue healing index; VAS, visual analogue scale.

^a^ Mean (standard deviation).

^b^ Proportion of patients reporting/experiencing the specific parameter.

^c^ For all patients the chosen painkiller was ibuprofen (400 mg) except for patient #7, who chose instead the intake of metamizole sodium (500 mg).

**Figure 2:**
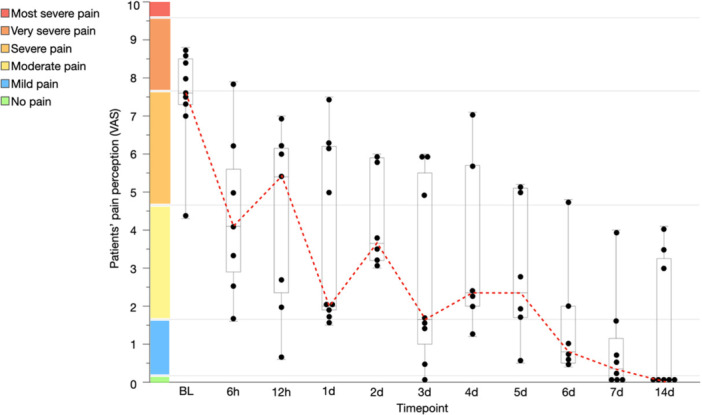
Patients’ pain perception assessed with the visual analog scale (VAS) and categorized from “no pain” to “most severe pain” at baseline (BL), 6 and 12 hours (h), and 1, 2, 3, 4, 5, 6, 7, and 14 days (d) after treatment. The interrupted red line connects the median VAS values at each time point.

### 3.2 Patients’ Pain Perception

Most of the patients (8/9 patients) reported prior to treatment severe or very severe pain. Within 1 day, all except for 1 patient reported a reduction in pain intensity, and 5 of them even a reduction to a score ≤ 2 (i.e., moderate pain). After 3 days 1 patient presented with no pain, 3 patients with mild pain, and a 5th patient with moderate pain, while the other 3 patients remained with severe pain. Pain scores mostly declined from day 4 to 6, and after 7 days 2 patients were pain‐free, 5 patients with mild pain, and 1 with moderate pain. At the final follow‐up after 14 days, 5 out of 8 patients reported complete pain resolution, while 2 patients reported a slight increase to moderate pain again and the pain of 1 patient slowly reduced but remained moderate.

The mean number of analgesics taken over the 14‐day period was 8.2 (4.9), but with clear interindividual differences. Five patients adhered to the recommended analgesic regimen for the first 2 days (i.e., 400 mg ibuprofen, 3‐times per day), while 1 patient (#7) chose to take metamizole sodium (500 mg, 3‐times per day), 1 patient (#9) refused to take any painkillers, 1 patient (#4) took only one painkiller each the first 2 days, and 1 patient (#1) took 4 painkillers the first day prior to dropping out from the study. After day 2, the additional number of painkillers taken until day 6 varied between 1 and 8 tablets, and none of the participants took any painkillers on day 7 or later. For details, see Table 1.

### 3.3 Soft Tissue Healing Index

At baseline, 6 out of 9 patients presented with a very poor or poor STHI. After 1 day, 6 patients showed an improvement, and after 3 days only 1 patient remained with a STHI of 2 (poor). After 7 and 14 days, 4 and 5 patients, respectively, achieved a very good or excellent healing and, overall, a mean STHI of 4.0 (0.8). For details, see Table 1.

### 3.4 Bone Exposure

At baseline, 7 out of 9 patients presented at least one exposed bone wall, with 3 of them with the maximum of 4 exposed bone walls. A clear improvement was visible after 3 days with all patients presenting with no exposed bone wall, and this result was maintained at day 7. After 14 days, a single patient presented again a minimal exposure of a single bone wall, which was also related to recurring moderate pain. For details see Table 1.

### 3.5 Necessity of Re‐Treatment, Complications, and Drop‐Outs

**Figure 3:**
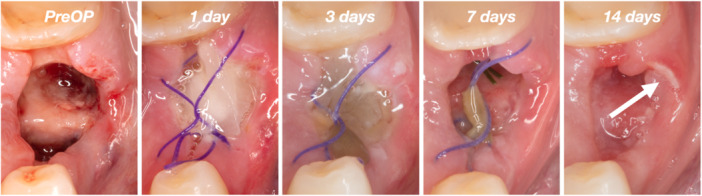
Wound healing sequence in patient #9. At baseline, the exposed bone walls are clearly visible, while after 1 and 3 days the socket entrance is covered with the collagen sponge. At day 7 and 14, the socket is completely covered by soft tissue, indicating a good healing process, which is also represented in low pain levels. However, at day 14 the pain level increased again due to a bone sequestrum, which penetrated the soft tissue at the bucco‐distal aspect (indicated with a white arrow) and was removed in the same session.

One patient received standard re‐treatment 3 days after the initial therapy due to an increase in pain level, and in a second case a bone sequestrum was removed after 14 days (Figure 3). One patient, who expressed disappointment due to persisting severe pain, chose to withdraw from the study after 1 day. For details see Table 1.

### 3.6 Patient Perspectives on the Treatment

Three out of 8 patients denied that they would pay anything extra for the adjunctive therapy; these 3 patients achieved a reduction of initial pain, however, still reported moderate pain at the last follow‐up after 14 days. The other 5 patients indicated that they would pay an additional fee of approximately € 50 to € 300 in case they would need AO treatment again. For details see Table 1.

## 4 Discussion

To the authors’ knowledge, this is the first clinical report on a xHyA gel combined with an absorbable collagen sponge as a treatment option for patients with AO. Most of the patients reported a relevant pain reduction after a single application of xHyA within the first 6 to 24 h, accompanied by advanced soft tissue healing within 3 days. Nevertheless, one patient dropped out of the study after 1 day due to persisting pain, and another patient required re‐treatment after 3 days due to recurring severe pain after 3 days.

In total, four studies were identified using HyA as an adjunct to irrigation and debridement of the socket in the treatment of AO (Dubovina et al. 2016; Suchánek et al. 2019; Hakobyan et al. 2020; Khachatryan et al. 2025); an overview of these studies is provided in Table 2. In all four studies, the treatment provided was repeated several times. More specifically, either a product with non‐xHyA was repeatedly applied daily (Suchánek et al. 2019), or every second day (Dubovina et al. 2016), while in the other 2 studies, magnetic laser therapy was repeated daily for 7 days after initial non‐xHyA application (Hakobyan et al. 2020; Khachatryan et al. 2025). In addition, these studies combined the application of non‐xHyA with other measures, for example, with aminocaproic acid (ACA) (Dubovina et al. 2016), with a custom‐made absorbable sponge‐like medical device combining HyA and OCDA (Suchánek et al. 2019), or with magnetic laser therapy (Hakobyan et al. 2020; Khachatryan et al. 2025). Only one of the previous studies (Suchánek et al. 2019) assessed pain already within the first 24 h, indicating a slightly superior pain relief with the treatment performed herein. The mean pain VAS scores in the other studies, 2 and 3 days after HyA application, ranged from 3.5 to 5.1 and from 1.9 to 3.6 compared to 4.2 and 2.8 herein, respectively, so overall comparable; after 6 to 7 days the pain VAS scores were also overall comparable but maybe slightly higher herein, but all within a range of no to mild pain levels. Three of the previous studies (Dubovina et al. 2016; Hakobyan et al. 2020; Khachatryan et al. 2025) included a control group without HyA application. All three studies reported a significantly faster pain relief and/or healing in the test groups with HyA application compared to the corresponding control groups. In addition, whenever a comparison was possible, the control groups without HyA of these three studies reported higher numbers compared to the data reported in the present study; for details, see Table 2.

**Table 2:** Overview of applied AO treatments, types of HyA, and results of investigated parameters in the present and previous studies.

Study (year) Study type Patient health status	HyA treatment (patient no.)	HyA type	Parameter		PostOP (any statistically significant difference to the control group without HyA is indicated with b)								
	*Control treatment (patient no.)* a			BL	1 d	2 d	3 d	4 d	5 d	6 d	7 d	8 d	14 d
Present studyCase seriesHealthy	Curettage, irrigation, xHyA and collagen sponge (9)	hMW, crosslinked	Pain (VAS 1–10)	7.5 (1.4)	3.8 (2.4)	4.2 (1.4)	2.8 (2.5)	3.5 (2.4)	2.9 (1.9)	1.6 (1.7)	0.9 (1.4)	NR	1.3 (1.9)
			STHI	2.0 (0.9)	2.8 (0.8)	NR	3.4 (0.8)	NR			3.6 (0.7)		4.0 (0.8)
	/		Bone exposure	2.2 (1.6)	0.8 (1.6)	NR	0	NR			0		0.1 (0.4)
			Re‐treatment	1/8									
Khachatryan et al. (2025)RCTDiabetic	Curettage, irrigation, HyA and magnetic laser (35)	Non‐crosslinked	Pain (VAS 1–10)	6.9 (0.1)	NR		3.6 (0.2)^b^	NR					
	*Curettage, irrigation (34)*			*7.1 (0.1)*			*5.9 (0.1)*						
			Soft tissue healing	Faster socket tissue healing with HyA									
			Re‐treatment	Not evaluated but daily application of magnetic laser only for 7 days									
Hakobyan et al. (2020)CTHealthy	Curettage, irrigation, HyA and magnetic laser (44)	Non‐crosslinked	Pain (VAS 1–10)	No pain in 93% of patients after 5 daily applications of magnetic laser therapy									
	*Curettage, irrigation (43)*		Soft tissue healing	Faster socket tissue healing with HyA									
			Re‐treatment	Not evaluated but daily application of magnetic laser only for 7 days									
Suchánek et al. (2019)case series (multicenter) Healthy	Irrigation, HyA, OCDA & calcium chloride (50)	Non‐crosslinked	Pain (VAS 1–100)	67.2 (20.6)	49.1 (25.6)	35.4 (25.3)	19.1 (20.8)	9.8 (5.5)	4.9 (11.4)	2.4 (8.2)	6.1 (6.4)	.NR	NR
	/		Re‐treatment	Not evaluated but daily application until ≤ 20 VAS									
Dubovina et al. (2016)RCTHealthy	Irrigation, HyA (10)	Non‐crosslinked	Pain (VAS 1–10)	7.3 (2.1)	NR	5.1 (2.5)	NR	2.4 (2.1)^b^	NR	0.7 (1.1)^b^	NR	0.2 (0.6)	NR
	Irrigation, HyA, ACA (10)			7.9 (1.7)		5.1 (2.5)		2.4 (2.1)^b^		0.7 (1.1)^b^		0.4 (0.8)	
	*Irrigation, Alvogyl (10)*			*7.4 (1.4)*		*7.2 (1.3)*		*5.1 (1.9)*		*2.9 (1.9)*		*0.8 (0.9)*	
	Curettage, HyA (10)			7.7 (1.5)		3.8 (2.7)		1.6 (1.5)^b^		0.3 (0.7)^b^		0^b^	
	Curettage, HyA, ACA (10)			7.9 (1.6)		3.5 (2.3)		1.8 (1.8)^b^		0.6 (1.1)^b^		0^b^	
	*Curettage, Alvogyl (10)*			*7.4 (1.7)*		*6.1 (2.6)*		*4.3 (2.5)*		*2.1 (1.9)*		*0.5 (0.9)*	
			Pain resolution	Significant less visits required until complete pain resolution in the groups with HyA									
			Re‐treatment	Not evaluated but applied every second day until pain resolution									

Abbreviations: ACA, aminocaproic acid; AO, alveolar osteitis; BL, baseline; CT, controlled trial; d, day; hMW, high molecular weight; HyA, hyaluronic acid; no., number; NR, not reported; OCDA, octenidine dihydrochlorid; PostOP, postoperative; RCT, randomized controlled trial; STHI, soft tissue healing index; VAS, visual analogue scale.

^a^ The description and data of the control groups are presented in italics.

^b^ Indicates a significantly better outcome in the group using HyA compared to the corresponding control group.

Considering previous studies using other biologics, such as platelet‐rich fibrin (PRF) (Rastogi et al. 2018; Reeshma and Dain 2021; Sharma et al. 2017; Keshini et al. 2020; Chakravarthi 2017), advanced‐PRF (A‐PRF) (Yüce and Kömerik 2019), concentrated growth factors (CGF) (Kamal, Salman, Abdul Razak, Qabbani et al. 2020; Kamal, Salman, Abdul Razak, Samsudin et al. 2020), and platelet‐rich growth factor (PRGF) (King et al. 2018; Pal et al. 2013), as adjuncts to standard AO treatment, a similar or slightly inferior pain relief was in general observed compared to that achieved with the xHyA used herein. For instance, two studies using PRF and one study using A‐PRF and PRGF, respectively, reported mean VAS scores ranging from 4.0 to 5.2 at 1 day after treatment (Sharma et al. 2017; Rastogi et al. 2018; Yüce and Kömerik 2019; King et al. 2018). Further, as painkiller consumption might also affect the wound healing process, patients were instructed herein to take during the first 2 days the same type and number of painkillers (i.e., 3 × 400 mg ibuprofen per day). However, only 5 patients followed this recommendation, while among the other 4 patients one was a drop‐out, 2 did not require the prescribed amount, and 1 chose a different type of painkiller (i.e., metamizole sodium). The 2 patients taking fewer painkillers experienced already 6 h after the intervention a clear reduction to moderate pain levels, while the patients requiring > 10 painkillers in total appeared to have severe pain levels longer. However, due to the limited number of patients and only a single patient not taking ibuprofen, it is difficult to draw any firm conclusions to which extent the intake of a non‐steroidal anti‐inflammatory drug might have affected the results.

The idea of using HyA in the treatment of AO is based on various aspects. More specifically, it has been described that endogenous HyA binds to fibrin and contributes to the organization of a 3‐dimensional matrix in the early stages of blood clot formation and wound healing (Weigel et al. 1986). Hence, the exogenous application of highly hygroscopic HyA probably enhances blood clot stability, which represents a relevant initial step of the wound healing process. Moreover, several in vitro studies have shown a positive effect of hMW HyA on cellular behavior critical to wound repair, such as the enhancement of the function of endothelial cells and fibroblasts, probably leading to improved angiogenesis and formation of granulation tissue (West et al. 1985; Gao et al. 2008; Asparuhova et al. 2019; Pilloni et al. 2025). In addition, HyA might interfere with numerous molecules responsible for pain perception and signaling (Marinho et al. 2021; Caires et al. 2015; Campo et al. 2012). For example, by interacting with cluster of differentiation (CD44) cell receptors, hMW HyA decreases the production of proinflammatory cytokines, such as interleukin‐1 beta (IL‐1β), tumor necrosis factor alpha (TNF*α*), and interleukin‐6 (IL‐6), and by inhibiting nuclear factor kappa‐light‐chain‐enhancer of activated B‐cells (NF‐kB) signaling pathway and downregulating transient receptor potential vanilloid 1 (TRPV1), it directly reduces nociceptor sensitization and pain transmission (Marinho et al. 2021; Caires et al. 2015; Campo et al. 2012). In this context, HyA seems to exert different biological effects depending on its molecular weight. Low molecular weight HyA can act pro‐inflammatory, whereas hMW HyA is associated with anti‐inflammatory and immunosuppressive properties (Fouda et al. 2016; Rayahin et al. 2015; Dovedytis et al. 2020). Hence, cross‐linking of HyA molecules might not only enhance time of residence at the application site but also amplify the anti‐inflammatory response, contributing to pain relief and an improved wound healing process. Finally, preclinical experiments reported improved extraction socket healing with the application of HyA compared to control groups without HyA (Mendes et al. 2008; Sá et al. 2013; Kim et al. 2019, 2016; Lee et al. 2021).

While pain levels can be seen as the most relevant parameter in AO treatment, despite its subjective nature, the present study also evaluated objective parameters, that is, STHI and alveolar bone exposure. At baseline, 60% of patients presented with poor or very poor STHI, and 7 out of 9 patients exhibited exposure of at least one alveolar bone wall. Following the AO treatment with xHyA and an absorbable collagen sponge, both STHI and alveolar bone exposure improved substantially within 24 h. After 3 days, all treated sockets were fully covered with granulation tissue (i.e., no alveolar bone exposure), and only one patient continued to demonstrate a poor STHI. However, it should be kept in mind that the collagen sponge may have masked any remaining alveolar bone exposure on day 1 and 3 (see Figure 3); in any case, the coverage of the alveolar bone with soft tissue was clearly evident latest on day 7. Two of the above‐mentioned studies using PRF also reported on alveolar bone exposure prior to and after treatment (Sharma et al. 2017; Keshini et al. 2020); although a higher baseline bone exposure was noted in the PRF studies (Sharma et al. 2017; Keshini et al. 2020), it still appeared that the improvement observed on day 1 and day 3 post‐treatment was more pronounced in the present study. From a clinical perspective, the comparison between repeated applications or blood‐derived products and HyA is likely in favor of HyA, as it can be seen as a rather time‐efficient treatment option, that is, its application, as described herein, requires only a few additional minutes, primarily for preparing the collagen sponge with HyA and with no need for multiple visits or for blood withdrawal, centrifugation equipment, and corresponding education of the personnel.

AO can be a highly painful condition that significantly impairs patients’ quality of life and often prompts the need for emergency treatment; as noted herein, 8 out of 9 patients reported severe or very severe pain at baseline. The costs for dental care in general and specifically emergency treatment vary a lot throughout Europe and are strongly dependent on the health care system. For example, in the United Kingdom the mean financial burden for the patients of emergency treatment is approximately € 100, with a maximum of about € 165 (Edwards et al. 2023), while in Austria emergency treatment in general and specifically for AO is fully covered by the public insurance system. Hence, it was interesting to see that 5 patients were willing to pay an additional fee of up to € 300 should they require the same treatment again. In fact, 4 out of these 5 patients would pay about € 100 or more, which would cover approximately the material costs for the presented treatment (i.e., 1 ampule of xHyA gel and 1 collagen sponge). However, the willingness to pay appears closely associated with treatment success, as the 3 patients denying paying any additional fee were those patients still reporting moderate pain at the final follow‐up.

However, some limitations should be acknowledged. Due to the lack of a control group, no assumptions can be made whether the observed effect is due to the xHyA gel, the collagen sponge, or the combination of both products; however, it may be speculated upon based on previous studies using a similar treatment approach, for example, in bone healing (Domic et al. 2023). For example, in preclinical studies, the combination of HyA with an absorbable collagen sponge in the treatment of infected extraction sockets of beagle dogs resulted in significantly higher bone formation compared to an absorbable collagen sponge only (Kim et al. 2019; Lee et al. 2021), indicating that HyA is the main effective component. The idea of using herein a collagen sponge is primarily based on maintaining space and additionally stabilizing the blood clot, which appears relevant in AO treatment due to the defect size and exposure to the oral cavity.

In conclusion, the present data suggest that a single application of xHyA combined with a collagen sponge may offer rather effective and fast pain relief, combined with a favorable healing process, in patients suffering from AO after tooth extraction. Furthermore, a minimal need for re‐treatment and favorable patient feedback (including willingness to pay) was observed.

## Author Contributions

**Danijel Domic:** patient selection and recruitment, data collection, data interpretation, manuscript drafting. **Peter Bauer:** patient selection and recruitment, manuscript drafting. **Nicola Ottenbacher:** patient selection and recruitment, data collection. **Kristina Bertl:** study idea, data analysis, data interpretation, manuscript drafting. **Christian Ulm:** study idea, manuscript revision. **Andreas Stavropoulos:** study idea, data interpretation, manuscript revision.

## Ethics Statement

The present project was approved by the ethics committee of the Medical University of Vienna (EK‐Nr: 1049/2021).

## Consent

Written informed consent was obtained from each patient prior to study inclusion.

## Conflicts of Interest

The authors declare no conflicts of interest. Andreas Stavropoulos is Editor‐in‐Chief and Kristina Bertl is Associate Editor of the journal Clinical and Experimental Dental Research.

## Permission to Reproduce Material From Other Sources

All material (i.e., figures and tables) was originally created by the authors; thus, no permissions are required.

## Supporting information

Supporting File

## Data Availability

The data that support the findings of this study are available from the corresponding author upon reasonable request.
